# Challenges in modelling the impact of frost and heat stress on the yield of cool-season annual grain crops

**DOI:** 10.3389/fpls.2025.1613432

**Published:** 2025-08-08

**Authors:** Jonathan Richetti, Victor Oscar Sadras, Di He, Brenton Leske, Pengcheng Hu, Yacob Beletse, C. Mariano Cossani, Ha Nguyen, Bangyou Zheng, David Matthews Deery, M. Fernanda Dreccer, Jeremy Whish, Julianne Lilley

**Affiliations:** ^1^ Commonwealth Scientific and Industrial Research Organisation (CSIRO), Floreat, WA, Australia; ^2^ South Australian Research and Development Institute, Adelaide, SA, Australia; ^3^ School of Agriculture, Food and Wine, The University of Adelaide, Adelaide, SA, Australia; ^4^ College of Science and Engineering, Flinders University, Adelaide, SA, Australia; ^5^ Commonwealth Scientific and Industrial Research Organisation (CSIRO), Acton, ACT, Australia; ^6^ The Department of Primary Industries and Regional Development, South Perth, WA, Australia; ^7^ Commonwealth Scientific and Industrial Research Organisation (CSIRO), Sta Lucia, QLD, Australia

**Keywords:** wheat, canola, barley, oat, chickpea, crop model

## Abstract

Frost and heat events at critical growth stages could cause large yield losses. These temperature extremes are increasing in frequency and intensity due to climate change in many parts of the broadacre cropping regions globally, presenting challenges to food production. For cool-season grain-growing regions, where summers are already too hot, heat and frost risks can limit adaptation options. Capturing these stresses in crop models accurately is increasingly important for evaluating the timing, severity, and yield consequences of extreme events. However, most existing process-based models were not designed to simulate short-duration temperature extremes, limiting their ability to assess climate risk and inform adaptation to frost and heat. Yield responses to heat and frost are associated with pollen sterility, grain abortion, accelerated senescence, and grain filling. Six challenges limit current modelling approaches: (1) inadequate spatial and temporal resolution of extreme events, (2) threshold-based and non-linear crop responses, (3) interactions between phenology and management, (4) cumulative and interacting stress effects across development stages, (5) limited representation of genotype-specific sensitivities, and (6) reliance on daily temperature data. Addressing these challenges requires improved use of sub-daily climate data, incorporation of physiological damage mechanisms, and enhanced crop- and genotype-specific parameterisation. These developments are critical for improving crop yield predictions under extreme temperatures in the context of climate change.

## Graphical Abstract

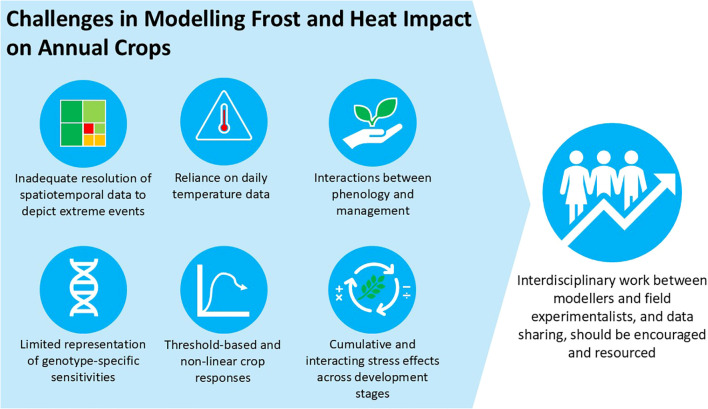

## 1 Introduction

Process-based crop simulation models simulate phenological development and aspects of the water, carbon and nitrogen economies of the crop (Boote et al., 1998; Holzworth et al., 2014; Ritchie et al., 1984; Stöckle et al., 2003). They operate at a daily time-step and are widely used to evaluate crop responses to the environment and management practices. Their predictive reliability often reflects the depth of empirical research invested in understanding crop response to abiotic stress, which tends to be better for crops such as soybean, rice, wheat, and maize than for pulses (Hao et al., 2021; Lake et al., 2021). The simulation accuracy of these models is also process-dependent, reflecting variability in both biological complexity and the degree to which the underlying mechanisms are understood. For example, we are better able to simulate crop-soil water balance (Cammarano et al., 2016; Gaydon et al., 2017), in which the underlying biophysics are well established (Penman, 1948), than biologically intricate processes, such as plant dry matter allocation, where modelling relies on coarse empirical partitioning factors because physiological understanding is lacking (Deng et al., 2020; Du et al., 2020; Ratjen et al., 2016; Weiner et al., 2009). Collectively, crop models typically achieve an r^2^ ~ 0.7 in large-scale comparisons of observed and modelled yield, which is sufficient to address many agronomic questions where resource supply is the main source of variation, such as the response of yield to rainfall or nitrogen availability (Asseng et al., 2001; Richetti et al., 2024; Saseendran et al., 2004). Crop models can model different species, as well as cultivar differences (especially phenology), but some environmental stresses, such as the impact of frost and heat on yields, are not modelled.

Early crop models designed for agronomic applications did not explicitly account for the effects of heat and frost stress on crop yield (Barlow et al., 2015). Incorporating these temperature stress effects expands the model’s realm of applicability. For example, augmenting a wheat model with heat stress functions led to a 73% reduction in yield prediction error (RMSE reduction of 1.8 t/ha; Liu et al., 2020). Another example is that by incorporating frost and heat stress response functions into a canola model, reduced the RMSE by 0.2 t/ha (Lilley et al., 2018). In chickpeas, although a cold temperature threshold has been proposed, no dynamic frost response function has been incorporated, limiting the model’s utility in risk assessment (Anwar et al., 2022). Optimal pairing of variety and sowing dates to minimise the combined risk of drought, heat and frost stress is a prime example of applications that require models that account for extreme temperatures (Barlow et al., 2015; Cammarano and Tian, 2018; Flohr et al., 2017; Hunt et al., 2019; Lilley et al., 2019). Extreme temperature events such as frost and heat pose a significant threat to plant metabolism, development, growth, and yield. Their impact depends on their timing, severity, and duration, and they can jeopardise local agricultural economies and global food security (Frederiks et al., 2015; McKersie, 2015). In 2024, widespread frost events in the Riverina region of Australia caused yield losses of up to 90% in canola, wheat, and barley crops, compounding drought-related stress and prompting growers to cut affected crops for hay (Smith, 2024). A late spring frost in Europe in 2017 resulted in economic losses of €3.3 billion (~US$3.8 billion) (Faust and Herbold, 2018). These increasingly frequent extremes add to the pressure on the major cropping systems due to anthropogenic climatic risks occurring earlier than previously anticipated (Jägermeyr et al., 2021). In the US, the combined effect of heat waves and droughts between 1980 and 2004 led to losses of more than US$125 billion, losses sixfold greater than drought alone (<US$18 billion) (Mittler, 2006). It is important to quantify the impact of extreme temperatures, including frost (resulting from minimum temperatures) and heat stress (resulting from maximum temperatures), on grain yield.

Grain yield in annual crops correlated primarily with the number of grains per unit area (Sadras, 2021), which is established during a species-specific developmental window known as the critical period (Carrera et al., 2024; Dreccer et al., 2018; Hunt et al., 2019; Kirkegaard et al., 2018; Sadras and Dreccer, 2015). This window is centred around flowering in cereals, and is centred at podding in canola and pulses (Dreccer et al., 2018). In variable climates, such as those found across broadacre cropping regions in Australia, the United States, Canada, Russia and South America, late-spring frosts during flowering and early grain-filling remain a significant risk (Zheng et al., 2015). Hence, pairing variety phenology and sowing date is a key management approach to managing the risk of yield loss to temperature extremes during this critical period (Chen et al., 2020; Flohr et al., 2017; Hunt et al., 2019; Lilley et al., 2019). These agronomic strategies become more important as climate change is projected to intensify the frequency and unpredictability of extreme temperature events (Lamichhane, 2021).

Numerous reviews have documented the physiological responses of plants to frost and heat stress (Farooq et al., 2011; Fischer, 2011; Frederiks et al., 2011; Grossman, 2023; Hasanuzzaman et al., 2013; Lara et al., 2025; Maqbool et al., 2010; Martino and Abbate, 2019; Sánchez et al., 2014; Younis et al., 2020). However, most insights are from studies at lower levels of biological organisation. These include alterations of gene expression, enzyme activities, photochemical reactions, carbon metabolism, metabolites/ion balance changes, stomatal conductance, chlorophyll content, photosynthetic efficiency, membrane stability, lateral diffusion of lipids, damage in cellular structures, etc (Bañon et al., 2004; Cheong et al., 2019; Hasanuzzaman et al., 2013; Kasuga et al., 1999; Levitt, 1980; Marcum, 1998; Mittler, 2006; Ruelland and Zachowski, 2010; Seetharam et al., 2021; Stitt and Hurry, 2002; Thakur et al., 2010; Theocharis et al., 2012; Thomashow, 1999; Vaultier et al., 2006; Vogg et al., 1998; Wise et al., 2004). While valuable, such perspectives offer limited insight into the impact of frost or heat stress on yield at the canopy or field scales. Bridging this gap requires scaling physiological responses into model-relevant traits such as leaf area index, radiation use efficiency or harvest index.

Several studies have investigated temperature stress impacts on yield in a crop modelling context. Barlow et al. (2015) focused on grain number around anthesis in wheat. Kogo et al. (2019) found only one maize statistical model (not process-based) that explicitly accounted for frost and heat stresses; Hao et al. (2021) reported considerable uncertainty in simulating wheat yield using APSIM Classic under frost, heat, nitrogen and water stress. Lake et al. (2021) identified five key challenges in modelling of frost and heat effects: (i) These events, though biologically and agronomically important, are statistically complex and rare; (ii) Field experiments face practical limitations under realistic stress conditions; (iii) Intra-field phenological variation differs with species, cultivar, management, and environment; (iv) Discrepancies exist between meteorological and canopy-level and finally, (v.) Crop responses to stress depend on the complex interplay between event timing, intensity, duration, recurrence, acclimation and recovery after the event. Arguably, both issues (i) and (iii) are in essence the same, the lack of adequate data. Another issue is that by adding more processes in the modelling framework, the model complexity increases and, consequently, the model’s equifinality also increases (Beven and Freer, 2001; Pasley et al., 2022). Which also contributes to the difficulty in implementing and publishing these improvements.

In light of these challenges, a focused synthesis of temperature stress representation in crop models is timely. This review centres on cool-season grain crop species and does not address intraspecific variation for which data is limited. Yield loss from late-spring frosts is a significant risk during flowering and early grain-filling in many temperate crop production areas worldwide. While frost damage during flowering is unlikely in summer-growing regions, heat stress remains a major concern. Our objective is to critically examine how crop simulation models represent the effects of extreme temperatures and to identify priorities for improving model responsiveness to temperature stress. Highly detailed models that prioritise physiological processes, such as Defraeye et al. (2014) and Zhu et al. (2014) operate at the expense of agronomic relevance and are beyond the scope of this review.

## 2 Modelling assumptions and gaps

First, we acknowledge that models are based on empirical relationships that represent the physiological processes of crop growth and development, and are validated against real-world data. Therefore, the accuracy of the models, in most cases, will depend on the availability of empirical research to develop the algorithms. Most process-based models do not account for several key physiological processes, such as acclimation – a crop’s adjustment to gradual changes in stress, compensation – growth recovery from suboptimal conditions, pathogen-induced freezing, and psychrophiles – cold-loving microbes that influence rhizosphere dynamics. Moreover, models do not explicitly explain interactions between extreme temperatures and other environmental factors such as solar radiation, water supply and demand, and nitrogen availability. While some of these interactions emerge depending on the model architecture, their representation is rarely mechanistic or generalisable. Lastly, it is important to note that abiotic stresses such as frost, heat, and drought are likely to have combined effects on yields that are not simply additive (Chen et al., 2019; Mahrookashani et al., 2017; Prasad et al., 2011; Stone et al., 1995). For example, a meta-analysis revealed that crops (including barley, canola, rice, wheat, maize, soybean, chickpea, groundnut, pearl millet, lentil, and potatoes) subjected to heat or drought stresses applied as a single-stress factor displayed a 33 and 48% yield reduction on average, respectively. In contrast, the average yield reduction in response to a combination of drought and heat stress was 65% (Cohen et al., 2021). Such findings challenge the assumptions of most existing crop models, which typically treat stress effects independently or additively. Here, we describe several processes supported by empirical literature but not yet incorporated into current models. Building on this, emerging work suggests that combining physiological principles and mathematics could lead to more mechanistically grounded algorithms, enhancing crop models’ predictive and explanatory power (Yin et al., 2021). The following subsections examine a series of specific physiological processes that are currently oversimplified or absent in crop models.

### 2.1 Temperature setting

There is often a discrepancy between the temperature recorded at the meteorological station (meso-climate) used to drive plant processes in models and that experienced by the plant (phyllo-climate). For example, most Australian Bureau of Meteorology temperatures are measured at 1.2-1.5 metres above the ground in a Stevenson Screen, where air temperature is approximately 2.2°C higher than the ground-level temperature (Grey, 2014). This difference is variable and influenced by factors such as time of day, solar radiation, evaporative demand, soil water content, soil type, crop density and stubble retention, and small topographic and aspect differences (Dreccer et al., 2018).

This issue is particularly pronounced for frost events. Radiative frosts typically occur on clear, calm nights when cold, dry, air descends (Risbey et al., 2019). Under these conditions, the upper canopy of the crop is commonly 2–5°C colder than adjacent screened temperatures at 1.2 m (Frederiks et al., 2012). Additionally, extrapolating point-based minimum temperature across a field is complicated by topographical gradients (Dixit and Chen, 2011) and the stochastic nature of ice nucleation and freezing within plant tissues (Livingston et al., 2018). Therefore, further research is needed to better distinguish the roles of the air and tissue temperatures in reproductive damage and yield loss.

In contrast, spring heat conditions in regions such as the Australian grain belt are typically driven by advective heat carried by wind from inland regions, leading to relatively uniform temperatures across the landscape. The actual temperature experienced by the individual plant organs is largely dependent on cooling by transpiration and soil water availability (van Oort et al., 2014). Temperature differences can occur between canopy air, spikes, and leaves (Dreccer et al., 2019) with heat reduction reported in non-drought conditions.

To develop relationships between crop damage and temperature extremes, it must be recognised that sensitive plant organs experience different temperatures from those recorded in standard weather stations. Currently, crop models rely on air temperatures from stations or grids derived from the stations, such as NASA Power (NASA, 2023) or the Australian Climate Grid Data (Evans et al., 2020). Snow cover further alters the temperature experienced by the crop relative to a screen; however, this is not an issue for already elongated crops during anthesis and flowering, and its effect is outside the scope of this review. Among major crop models, APSIM and WOFOST do not account for snow, and DSSAT includes it simplistically as part of the soil water balance, while STICS simulates snow accumulation, melting, and its insulating effects on soil temperature.

To calculate temperature data across a day, crop models fit daily maximum and minimum temperatures to a curve. For example, APSIM generates temperatures at 3-hour intervals (Holzworth et al., 2018, 2014), while DSSAT fits temperatures at hourly intervals (Hoogenboom et al., 2019). Nonetheless, the models rely solely on observed maximum and minimum temperatures as inputs; that is, the 3-hour or hourly temperatures used in the internal calculations are fitted and not observed temperatures. This approach limits the ability of the model to capture the duration of temperature-related stress. While it is not currently possible for global gridded data sources to provide hourly data, ideally, models would use hourly recordings from modern weather stations instead of fitted values. While the duration of exposure to temperatures above or below the threshold is important, most models overlook duration to achieve model simplicity. Models use temperature thresholds for the functions; however, literature often reports cardinal temperatures. We further explore this critical issue in Section 4.

### 2.2 Acclimation

Acclimation influences the plant phenotype in response to extreme temperatures (Charng et al., 2023; Islam et al., 2018; Janská et al., 2010; Juurakko et al., 2021; Müller and Rieu, 2016). Some models (e.g. GECROS) considered cold acclimation via enzyme kinetics or adjusting photosynthetic and respiration efficiency based on temperature history (Hikosaka et al., 2006). Modelling acclimation to low or high temperatures presents challenges due to the complex physiological and biochemical processes involved, which are often genotype-specific and interact with other environmental factors. Additionally, limited experimental data and the need to scale molecular responses to whole-plant and canopy scales further complicate the quantification of acclimation in crop models (Atkin and Tjoelker, 2003; Wang et al., 2017). For low temperatures, full expression of plant tolerance occurs only in the vegetative phase whereas plants in the reproductive phase have a limited ability to acclimate (Fowler and Limin, 2004). Hence, the assumption of lack of acclimation to low temperature is unlikely to bias the prediction of yield response to frost damage at the reproductive phase.

In contrast to frost, acclimation to heat stress before flowering can persist after flowering. In pot-grown wheat, 2-day exposure to 32/28°C twice, at the seven-leaf and the nine-leaf stages, improved the tolerance to heat (34/30°C) up to two weeks after anthesis (Wang et al., 2011). Acclimated plants were superior to their non-acclimated counterparts for metabolic and gas exchange traits, and these low-level traits scaled to higher starch content in kernels (Wang et al., 2011). Hence, the assumption of no acclimation to heat stress may have consequences for the modelling of yield response to heat stress.

Vernalisation and acclimation are different responses to low temperature; they occur simultaneously yet independently. In cereals, the vernalisation response has been linked to vegetative frost tolerance (Fowler et al., 1996), but vernalisation and acclimation have been shown to be distinct processes that respond differently to low temperatures (Bond et al., 2011). In canola, vernalization is also unrelated to cold tolerance, and both spring and winter types have similar cold tolerance and capacity for cold acclimation (Trischuk et al., 2014). This review does not make further consideration of vernalisation as it is not directly associated with damage to crop yield.

### 2.3 Compensation and competition

Agronomically important compensation occurs at two levels: individual plant and population. The decline in the phenotypic plasticity of yield components with ontogeny (Sadras and Slafer, 2012) reduces the ability of individuals to compensate for damage caused by extreme temperatures later in the growing season (Barlow et al., 2015). At the plant-level, compensation may include grain-setting in lower-hierarchy positions on wheat ears to offset grain loss in dominant positions, larger grain size compensating for reduced grain number, and increased fertility of tillers; some of this has been documented experimentally (Leske, 2021; Marcellos and Single, 1984). In indeterminate crops like canola and pulses, unaffected cohorts of flowers or seeds can compensate for stress-related losses by increasing grain set or grain size. Grain number can be significantly reduced when frost or heat stress occurs during the critical period around flowering in cereals or podding in pulses and canola (Sadras and Dreccer, 2015). In wheat, grain weight is affected from pre-anthesis when carpels are formed, through to grain filling (Calderini et al., 1999; Hasan et al., 2011). In canola, grain weight and oil content are affected by stress during the critical period (Kirkegaard et al., 2018).

Current models do not account for intra-specific competition for resources such as light, water, and nitrogen, nor do they consider genotypic variability. Small grain cereals can compensate between tiller number and grain size, and indeterminate crops through increased branching. Thus, modelling population-level compensation requires accounting for plant-plant hierarchies in heterogeneous stands, which are relevant to field crops but beyond the scope of the models considered in this study.

### 2.4 Uniform stands

Crop models typically estimate a single date for a given phenological event, with few exceptions. However, the spread of phenology among plants and plant parts, such as tillers and branches, is important for the crop-level response to extreme events. This variation depends on species, cultivar, management practices, and environment (Borrás and Vitantonio-Mazzini, 2018; Crone et al., 2009; Masino et al., 2018). For example, the duration of flowering, from its onset to 50% completion at plot level, varied 3-fold across a set of 292 rice lines (Bheemanahalli et al., 2016). Indeterminate crops, such as canola and soybeans, are ultimately simulated as determinate crops.

Most process-based models assume plant populations are uniform stands, overlooking hierarchies in plant size and variation in phenological development. The assumption of the uniform stand may underestimate the spatial variation in stress exposure at canopy and field scales, which has significant implications for yield prediction. In contrast, functional-structural plant models address these limitations by simulating competition. They account for individual plant differences by simulating microclimatic differences within canopies and adjusting resource acquisition and allocation among organs based on plant positioning and biomass. Such models have the potential to consider variation in stress exposure (Fourcaud et al., 2007; Vos et al., 2010).

### 2.5 Spatial variation

Spatial variation commonly occurs during extreme weather events, often influenced by terrain aspects such as elevation and slope (Dixit and Chen, 2011; Gobbett et al., 2021, 2020). Crop models have often been designed for a specific scale, corresponding to the scale of the processes they seek to predict. Also, evaluation of spatialised predictive crop models depends on the number, location, and spatial footprint of validation data (Pasquel et al., 2022). Thus, the use of point-scale models to answer spatial questions, such as sub-paddock analysis of frost and heat impact on yields, will require climate, soil and crop data at the multi-point scale. For example, data such as the survival of plants in damaged areas, the spatial distribution and extent of the damage, and the duration and intensity of frost and heat events, become crucial for establishing and validating temperature/damage responses.

### 2.6 Other factors not accounted for by crop models

The roles of pathogen-induced freezing and psychrophiles in plant responses to low temperature have been reviewed by (Juurakko et al., 2021). While not all ice-nucleating bacteria are pathogenic, those that are can cause ice propagation that ruptures plant membranes, allowing bacteria access to intracellular nutrients (Gusta and O’Connor, 1987; Lindow, 2023). The interaction between direct frost damage and indirect damage caused by psychrophilic bacteria and fungi could be important, but is beyond the scope of current models. The process of heat transfer between the flower, glumes, and leaf sheath involves more than conduction (Martino and Abbate, 2019); however, their model only accounts for conduction.

## 3 Modelling approaches

Models used different approaches to simulate the effects of extreme temperatures on crops. Some models include functions that impact crop survival, leaf area index, grain number, grain weight, and overall yield. When scaling plant population density and leaf area index, the effects of extreme temperatures are transferred to yield via the capture of resources such as radiation and water. Scaling of yield and yield components is usually direct, based on empirical “penalty” functions. Overall, these are scalars (0–1) applied as a multiplier to a potential physiological rate, effectively reducing the rate to simulate crop responses under stress. In APSIM, modular penalty functions can be customised and can adjust photosynthesis, phenology and partitioning based on stress indices (Holzworth et al., 2018). In DSSAT, penalty functions are typically multiplicative stress factors applied to potential growth rates (Hoogenboom et al., 2019). In STICS, they are referred to as stress response functions and reduce processes like leaf area development, biomass production, and phenological progression (Brisson et al., 2003). In WOFOST, reduction factors affect assimilation rate, leaf area expansion, and transpiration (de Wit et al., 2019).

### 3.1 Crop survival

Most models have a kill switch, i.e., a threshold for lethal temperature, water stress, nutrient deficiency or a defined rule that terminates the crop. Regarding frost temperatures, models have a threshold where the plants are killed if the lethal temperature is reached at any growth stage before anthesis (Donatelli et al., 2010; Hanks and Ritchie, 1991; Hoogenboom et al., 2019; Williams, 1995). In cereals such as wheat and barley, subsequent regrowth could occur, producing a substantial yield. This yield, however, is likely penalised by the delay in development and growth and compounded by the effect of higher temperatures experienced later in the season. To improve accuracy, models need to account for this confounding effect of the combination of frost and hot temperatures.

The WOFOST model (de Wit et al., 2019) simulates the impact of cold stress on winter crops, which can experience frost during the winter dormancy period. It incorporates a winter-kill module based on the FROSTOL model, which simulates the hardiness (frost tolerance) of plants as a function of snow depth, temperature and vernalisation stage (Bergjord et al., 2008). The kill function in WOFOST estimates the fraction of plants dying due to the daily minimum crown temperature. However, the effect is reportedly difficult to validate or apply since the causal relationships are not clear, i.e. the survival of winter crops also depends on factors such as fungal diseases and asphyxiation, among others (Bergjord Olsen et al., 2018; de Wit et al., 2012) (see assumptions, section 2).

### 3.2 Leaf area

The actual leaf area is the net result of expansion (individual leaf, tillering, etc) and senescence. Leaf area damage functions focus on leaf senescence and overlook leaf expansion and tillering; this is a potential source of bias.

The APSIM-Nwheat model (Asseng et al., 2011) hastens leaf senescence by multiplying the calculated senesced leaf area under normal development by a heat stress factor (Eq.). This factor increases threefold when the daily maximum temperature exceeds 34°C, and sixfold at 40°C. The heat stress function was based on glasshouse and field experiments which demonstrated senescence at temperatures above 34°C. However, neither data nor clear metrics of the observed *vs*. simulated LAI that test the senescence function described in Equation 1 are presented.


(1) $$\begin{array}{c} F_{heat}=4-(1-(T_{max}-34)/2)\\ F_{heat}=1\;T_{max}\leq 34^{o}C \end{array}$$

In APSIM Classic (Holzworth et al., 2014) LAI is reduced by a stress-induced leaf senescence factor, see Equation 2.


(2) $$\text{Δ}LAI_{sen}=k_{sen}*LAI$$

Where $k_{sen}$ is the slope of the linear equation relating the daily minimum temperature for frost and daily maximum temperature for heat; which are defined by the senescence temperature and a factor that are linearly interpolated. For heat stress, $k_{sen}$ varies from 0 at 34°C to 0.4 for ≥ 45°C (www.apsim.info). The default $k_{sen}\;$ for frost is zero meaning that there is no frost stress impact on LAI. Insufficient data for testing remains an issue, and the acceleration of LAI senescence due to extreme temperature was discontinued in APSIM Next Generation.

The STICS model (Brisson et al., 2009, 2003) represents frost damage using indices ranging from 1 (no damage), to 0 (lethal frost). The temperature response varies depending on the developmental stage. The occurrence of frost at the seedling and plantlet stage reduces plant density, while frost before or after the juvenile phases affects foliage by accelerating senescence. To the best of our knowledge, observed and simulated leaf area that validate the senescence as a function of extreme temperatures are not publicly available.

Tschurr et al. (2023), defined a Frost Damage Index (FDI) that accounted for the reduction in canopy cover (effectively leaf area) of wheat in response to cold temperature stress during the vegetative phase.

### 3.3 Grain number and size

Crop models account for grain number and/or size losses due to extreme temperatures in various ways, though few account for genotypic variability. In APSIM-Nwheat (Asseng et al., 2011), dry matter accumulation in the grain is driven by the potential accumulation per kernel, which depends on the balance between supply and demand for assimilate. For single kernel growth, dry matter accumulation is calculated based on mean temperature when it is below 10°C and on maximum and minimum temperature amplitude when above 10°C. Extremely high maximum temperatures reduce the potential kernel growth rate demand. The model caps maximum grain weight at 55 mg/kernel. Comparisons of observed and simulated data are presented in plots of (a) reduction in kernel weight per degree increase in temperature across temperature ranges and (b) grain yield loss per day above 34°C, with no metrics such as RMSE or R^2^ provided (see Asseng et al., 2011).

The STICS model reduces grain number as a function of frost temperatures during flowering and grain filling stages (Brisson et al., 2009, 2003). The model penalises grain number similarly to the method used for frost damage to LAI, via a 0 to 1 multiplier. As for accelerated leaf senescence, the data underpinning the indices and equations are not readily accessible and not reported in the documentation (Brisson et al., 2009, 2003). Furthermore, the relationship between these losses and grain filling is not clear.

Martino and Abbate (2019) used field data to model grain number reduction in response to timing, intensity, and duration of frost events. The algorithms ensure that successive frosts only affect florets which were not damaged in a previous frost. Frost damage at the crop level is estimated from the sum of damage at the spike level weighted by the frequency of spikes in each developmental stage according to the normal distribution. However, to integrate such functions into daily-time step models, a link between the duration of the frost event, measured in minutes, and crop models would be required.

### 3.4 Yield

A daily yield reduction factor, based on the development stage and daily air temperature was applied in APSIM Classic to simulate the impact of frost stress on wheat yield (Bell et al., 2016, 2015; Zheng et al., 2015). Yield reductions were calculated for each day and multiplied so that increasing numbers of stress events cumulatively reduced the yield (**Table 1**). Zheng et al. (2015) modelled the distribution of exposed heads at susceptible post-heading stages using a trapezoidal variable yield multiplier, ranging from 1 (no yield reduction) to 0.1 (90% yield reduction) across developmental stages from boot-swollen (Z45) to fully ripe (Z89). Bell et al. (2015) validated heat damage functions against yield reductions reported by Farre et al. (2010). This approach penalised yield post-simulation, making it practical but not process-based. Consequently, the approach does not simulate the impact of frost and heat stress on grain development by affecting grain size or grain weight, but rather reduces the final yield.

**Table 1:** Wheat minimum and maximum daily temperature criteria for frost and heat stress during sensitive growth stages (Zadoks growth stage; (Zadoks et al., 1974) and estimated yield reductions (Bell et al., 2016).

Stress	Level	Daily min or max temperature (°C)	Zadoks sensitive stage	Yield reduction per day (%)
Frost	Mild	0 to 2	Z60-69	10
	Moderate	-2 to 0	Z60-75	20
	Severe	< -2	Z60-79	90
Heat	Mild	32 to 34	Z60-79	10
	Moderate	34 to 36	Z60-79	20
	Severe	> 36	Z60-79	30

Similarly, in canola, a yield multiplier (**Table 2**) depending on the daily temperature during a sensitive period was applied (Kirkegaard et al., 2016; Lilley et al., 2018, 2015). Yield reductions were calculated for each day and accumulated (multiplicatively), resulting in cumulative yield reductions as the number of stress events increased. The sensitive period to frost and heat stress in canola spans approximately 6 weeks during the flowering and early pod-filling stages. The specific thermal time period relative to the appearance of the first flower is detailed in **Table 2**.

**Table 2:** Canola minimum and maximum temperature criteria for frost and heat stress during phenologically sensitive stages and estimated yield reductions (reproduced from Lilley et al., 2015).

Stress	Level	Daily temperature (°C)	Phenological stage	Yield reduction per day
Frost	Mild	0 to 2	140–800 degree-days after first flower (early pod-filling period)	2%
	Severe	< -2		10%
Heat	Mild	30 to 33	0–630 degree-days after first flower (flowering period)	10%
	Moderate	33 to 36		18%
	Severe	> 36		35%

The frost and heat multipliers were updated from step-wise damage function (**Table 2**) to linear and trapezoidal response functions, resulting in improved simulations with a root mean square error reduction of ~0.2 t/ha (Lilley et al., 2018). These updates were validated against additional experimental data comprising observations for 17 cultivars across 81 growing seasons in Australia. Further refinements in this paper reduced the simulation error of canola yield in APSIM Classic from 50% to 20% (NRMSE) and 0.8 t/ha to 0.3 t/ha (RMSE).

## 4 Air temperature thresholds

Chilling temperature has been defined as ‘any temperature that is cool enough to produce injury but not cool enough to freeze the plant’ (Levitt, 1980), while frost refers to ice formation on crops, usually when the air temperature falls below 0°C. Similarly, heat stress disrupts plant growth and development, particularly during reproductive stages, often leading to yield loss (Shah and Paulsen, 2003; Talukder et al., 2014). Conceptually, frost or heat temperature thresholds mark the critical temperatures where crop damage begins and peaks. These thresholds vary with crop species, cultivar, and development stage with lethal and supra-optimal temperatures differing accordingly. Determining temperature thresholds for heat and frost requires trait *vs* temperature data, fitted response functions, and inflection points accounting for effects of genotype, environment, management, and their interactions. Several experiments have aimed to develop response functions for crop models. For example, studies on wheat have shown clear reductions in grain number and weight due to brief periods of high-temperature stress (Elía et al., 2018; Prasad et al., 2014). Delahunty et al. (2023) tested lentil response to high temperature (>32°C) using late sowing and partial shading. A field study comparing frost-exposed and frost-protected wheat showed a yield reduction from 2.6 to 1.8 t ha^-1^, partly due to a decrease in grain number per spike from 29 to 19 (Ferrante et al., 2024). These experimental designs could be adapted for studies of other crops.

While field studies often report significant damage (e.g. from ANOVA), the inherent temperature variability in field conditions frequently hinders the establishment of clear thresholds. Typically, temperature reporting includes (i) maximum and minimum temperatures and may also include (ii) duration above or below a particular temperature and (iii) the number of extreme temperature events based on a temperature threshold. However, it is the yield loss in the field that models aim to simulate, and field experiments, which capture acclimation and stress interactions, are ideal for model validation, particularly when phenotyping occurs at the level of the processes affected. For example, phenotyping studies with remote sensing that also assess losses due to frost (Nuttall et al., 2019) offer information valuable for modelling. That is, if the model aims to simulate yield loss by accounting for natural variability and interactions between stress, plants and environment, then accurate information on temperature ranges, thresholds and variation from field experiments is essential. Unfortunately, this foundational data is often inaccurate or incomplete.

**Figure 1:**
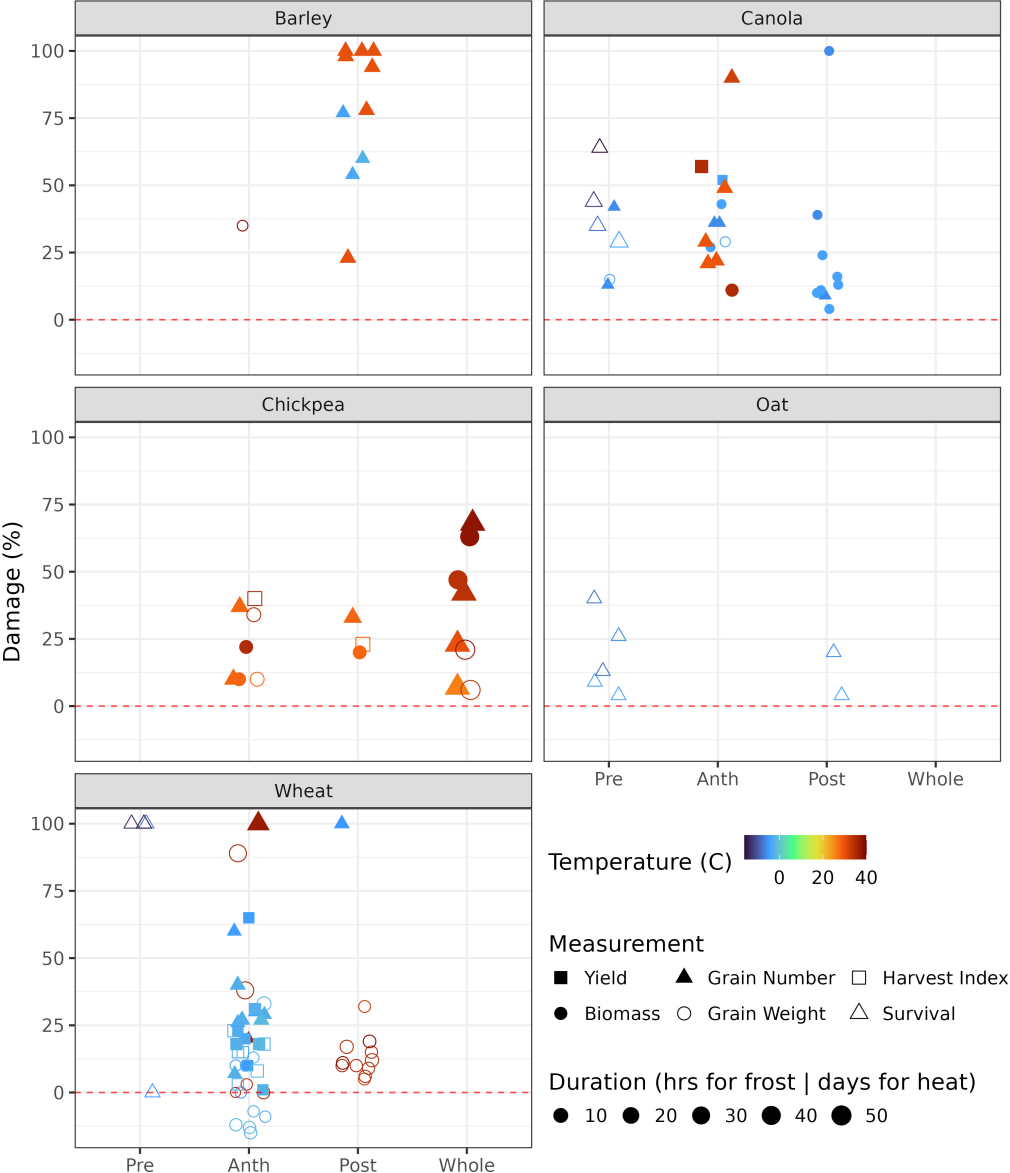
Frost and heat air temperatures at which significant damage was observed for six measured attributes for five crops. Negative damage means an increase in the attribute in response to stress; it only occurred for wheat grain weight in the Nuttall et al. (2019) study. Data sourced from various papers, see **supplementary table 1** (Blumenthal et al., 1994; Chen et al., 2019; Cromey et al., 1998; Fiebelkorn and Rahman, 2016; Fuller et al., 2007, 2007; Gusta and O’Connor, 1987; Hawker and Jenner, 1993, 1993; Hikosaka et al., 2006; Jenner, 1991; Jumrani and Bhatia, 2014; Kovaleski et al., 2020, 2020; Leske and Biddulph, 2022; Marcellos and Single, 1984; Morrison and Stewart, 2002; Nuttall et al., 2019; Polowick and Sawhney, 1988; Prasad et al., 2014; Randall and Moss, 1990; Savin et al., 1997; Stone and Nicolas, 1994; Wang et al., 2006). Pre, pre-anthesis; Anth, anthesis; Post, post-anthesis; Whole, whole growth period.

In this review, we examined the literature and have summarised and calculated relative damage or loss at various temperatures (**Figure 1**). For example, a grain weight reduction from 40 mg per kernel (control) to 35 mg per kernel (frost/heat treatment) represents a 12.5% loss (1 – 35/40). Total plant or organ kill is assumed to be 100% damage. The inconsistency on reporting prevents an overall summary of yield loss; thus, we aggregated the percent damage on yield-related variables, such as survival, biomass, harvest index, grain weight, grain number (spikelets killed), and final yield. We have summarised a range of temperatures and their impacts across various development stages and organs in different crop species. The various stages where treatment was applied were split into pre- and post-anthesis, anthesis, and the whole plant growth period. Results of the analysis appear in **Supplementary Table 1** and are summarised graphically in **Figure 1**. Experimental environments were not clearly split in **Figure 1** (see **supplementary materials** for further detail), but it is noted that data from chambers and glasshouses often fail to represent field conditions accurately and need to be interpreted with caution. Field data are more realistic but still face challenges in the removal of confounded environmental effects, such as the association between low vapour pressure deficit and high temperature (Bonada and Sadras, 2015). Methods for measuring frost and heat events across crops are not consistent, and this also reduces the comparability of the data between crops and experiments.

Abiotic stresses culminating in yield reduction are associated with flower abortion (reducing grain number) and grain damage (reducing grain quality and weight). These observations help define the critical period, defined as the developmental stages when crop yield is most susceptible to stress events such as frost and heat: see Dreccer et al. (2018) for cereals and pulses, Lake and Sadras (2014) for chickpea, Monzon et al. (2021) for soybean, Kirkegaard et al. (2018) for canola, and Sadras and Calderini,. (2021) for other major crops. For heat tolerance during flowering in rice, wheat, and maize, see (Liu et al., 2023). Models that capture this time-sensitivity to damage will more likely accurately capture yield losses due to frost and heat.

## 5 Discussion

In his 1980s summary of state-of-the-art in crop modelling, Monteith (1981) concluded that “Progress has not been impeded by lack of ingenuity in model building but rather by the large number of grey areas in our physiological knowledge which modellers have drawn attention to”. This observation remains relevant to the modelling of extreme events in annual crops, for which physiological understanding continues to be a bottleneck. General response functions for setting of maximum grain number pre-flowering and grain size post-flowering appear valid across a range of crops, not only wheat as previously proposed (Barlow et al., 2015). However, deriving the temperature thresholds and response functions for occasional, extreme temperature stress events remains challenging, as experiments designed to capture the required data for modelling are still needed. Most experiments on frost and heat stress are designed for other purposes. For example, studies on barley show significant impacts on yield and other traits (Högy et al., 2013), but these treatments compare elevated *vs*. ambient temperature making it impossible to define a stress threshold. Furthermore, damage is often assessed visually rather than quantitatively. For example, in a scoring system by (Fiebelkorn and Rahman, 2016), 0 denoted dead, 5 denoted no damage, and scores of 1–4 were based on visual estimation of frost damage. A lack of detailed data hinders objective improvements to crop models. Nonetheless, some studies provide valuable data for developing response functions. For example, Nguyen et al. (2013) and Prasad et al. (2014) explored grain number and weight responses to the timing of high temperature stress, while Martino and Abbate (2019) conducted hypothesis-driven, data-rich studies. The advent of high-resolution phenotyping offers the opportunity to gather quantitative data that can be used in process-based modelling (Nuttall et al., 2019). Although the development of frost damage indices at broad scale through remote sensing (Wang et al., 2020) demonstrates potential to assess damage at a regional scale, such remotely-sensed indices do not necessarily model the processes. **Figure 1** summarises data that can facilitate the first steps in the development of response functions for various crops to frost and heat stress. However, a major limitation to modelling crop responses to extreme temperatures is our superficial understanding of the underlying physiological processes.

The current stress and heat multipliers are based on limited data without rigorous optimisation. Furthermore, the multiplier functions are linear, which may over-simplify the biological response. Significant research has been conducted at lower levels of biological organisation, focusing on the effects of frost and heat stress on physiological processes. These effects are only partially understood, particularly at the molecular and cellular levels, ranging from gene expression to short-term metabolic responses (Bañon et al., 2004; Cheong et al., 2019; Hasanuzzaman et al., 2013; Kasuga et al., 1999; Levitt, 1980; Marcum, 1998; Mittler, 2006; Ruelland and Zachowski, 2010; Seetharam et al., 2021; Stitt and Hurry, 2002; Thakur et al., 2010; Theocharis et al., 2012; Thomashow, 1999; Vaultier et al., 2006; Vogg et al., 1998; Wise et al., 2004). Scaling these findings to agronomically relevant processes used in crop simulation models remains a challenge. For this reason, empirical functions are employed to scale processes, but experimental data are insufficient to fully capture the combinations of stress intensity, duration, and timing needed to build robust response curves. While these studies are insightful, they fall short in relevance to agronomic traits such as yield formation over the growing season. This review outlines current knowledge to inform the parameterisation of development stage-based yield-damage functions, which can be integrated into process-based crop models.

Differences between air and canopy temperatures also present a gap that needs addressing. The Stevenson Screen effect, reported to be +2.2°C compared to ground or canopy temperature (Grey, 2014), introduces potential systematic errors in crop models as air temperature data from weather stations is a standard data input. However, since most studies report air temperature impacts on plants, the Stevenson Screen effect may already be embedded in model development. Nonetheless, land surface temperature, particularly from satellite imagery, could provide valuable insights into the spatial-temporal distribution of frost and heat events across landscapes. Satellite products offer better spatial resolution than gridded weather data (e.g. Himawari 8 resolution at 2 km *vs*. Australian Bureau of Meteorology at 5km). From a crop modelling perspective, methods that deliver finer spatial resolution for daily minimum and maximum air temperature are warranted.

Future developments in crop modelling should incorporate frost and heat response functions. We have expanded and updated the compilation of temperature-damage relationships for wheat (Barlow et al., 2015) to include other crops. In cereals, frost or heat stress during the critical period can reduce grain number due to stress effects on meiosis and flower abortion. Subsequent stress events after the potential grain number is set primarily affect grain weight or can result in grain abortion. Most damage functions simply penalise yield. To improve models, separate response functions could be developed for grain number and grain weight, each linked to developmental stage and temperature thresholds. Based on the assumptions presented in Section 2 and the temperature and damage percentages reported in Section 4, response functions for frost and heat stress could be incorporated into a wide range of crop models to enhance their accuracy under extreme temperature conditions. Additionally, stress-induced leaf senescence from frost and heat effects on foliage may contribute to yield losses by reducing LAI and thus limiting assimilate supply during grain filling. However, this hypothesis remains untested.

When developing and testing models with frost and heat yield reducing components, two key components need consideration; i) yield response to frost and heat events, which depends on the timing (on a phenological scale), intensity (how cold or hot, i.e. temperature) and duration of stress (time spent above or below temperature thresholds); and ii) validation of response functions using experimental data across diverse environments, crops, and genotypes. Furthermore, the development of frost and heat response function should be in the form of elegant and robust equations that achieve both biological rigour and model simplicity, and interact with other stress effects (e.g., heat and drought combined effect). This approach aims to bridge the gap between the understanding of the effects of frost and heat stress effects at the cellular and plant organ level and their agronomically relevant impacts at the field scale.

Lastly, the use of modelling thinking to inform experimental design will facilitate the development of crop models which capture the effects of frost and heat stresses on crop yields (Craufurd et al., 2013; Nuttall et al., 2019; Rötter et al., 2018). This begins with identifying the key physiological processes affected by frost and heat stresses, which are required to conceptualize the processes within a model. Key physiological processes related to reproductive biology include floret initiation, development and growth, and grain formation and abortion. Ideally, field experiments should incorporate diverse combinations of genotypes, environments, and management practices. Temperature-controlled field plots or open-top chambers can facilitate the imposition of frost and heat events alongside natural stress events to capture a range of responses across growth stages (Leske and Biddulph, 2022). Additionally, meteorological data (e.g. canopy temperature, humidity, and radiation) should be systematically recorded in the canopy with high temporal and spatial resolution. This data can help quantify the microclimatic differences and link environmental conditions to crop stress responses. Remote sensing technologies, such as thermal imaging and hyperspectral sensors (Deery et al., 2019), offer valuable tools to enhance data collection by capturing spatial and temporal variability in stress responses. However, field experiments are labour-intensive, and introducing additional requirements might not be feasible. Therefore, promoting data sharing in accordance with the F.A.I.R. principles (Findability, Accessibility, Interoperability, and Reusability) can help generate robust datasets to improve model development (see the example of plant traits in Kattge et al., 2020).

## 6 Conclusions

Current process-based modelling approaches face six major challenges: (1) insufficient spatial and temporal resolution for extreme events, (2) non-linear and threshold-based crop responses, (3) complex interactions between phenology and management, (4) cumulative and interacting stress effects throughout development stages, (5) inadequate representation of genotype-specific sensitivities, and (6) dependence on daily temperature data. Using sub-daily climate data, incorporating physiological damage mechanisms, and improving genotype-specific parameterisation will improve the modelling of frost and heat event impacts on the yield of cool-season annual grain crops. To achieve this, more interdisciplinary work between modellers and field experimentalists and data sharing should be encouraged and resourced.
